# A Unique Case of Adult-Onset Still’s Disease Not Responsive to Steroid Treatment

**DOI:** 10.7759/cureus.49492

**Published:** 2023-11-27

**Authors:** Davis Martin, Collin A Toups, Arya Loghmani, Barrett Ford, Vinod Dasa, Luis Marrero

**Affiliations:** 1 School of Medicine, Louisiana State University Health Sciences Center, New Orleans, USA; 2 Rheumatology, Louisiana State University Health Sciences Center, New Orleans, USA; 3 Orthopaedic Surgery, Louisiana State University Health Sciences Center, New Orleans, USA

**Keywords:** neutrophilia, autoinflammation, osteoarthritis, aosd, adult-onset still's disease

## Abstract

A 53-year-old patient was admitted to the emergency department, presenting with fever, generalized weakness, and various myalgias and arthralgias lasting over seven days. Based upon the patient’s worsening symptoms, elevated white blood cell count with neutrophilia and overall presentation, she was initially treated for an infectious cause and prescribed various antibiotics and antipyretic medications. As the patient’s condition continued to worsen throughout the initial days of her intake, she was tested for a variety of infections, including coronavirus disease 2019 (COVID-19), Streptococcus, and influenza, and was administered a viral respiratory panel, all of which resulted negative. Upon the development of an evanescent rash on hospital day 9, as well as other symptoms including sore throat, arthritis, and an elevated fever present for over a week, a rheumatology consult now expressed concern for a possible case of Adult-Onset Still’s Disease (AOSD). In line with the current treatment used for AOSD and the absence of all other infectious causes, the patient discontinued antibiotic treatment and was started on 125 milligrams of intravenous methylprednisolone every six hours. The patient showed minor improvements in symptoms over the next 24 hours but soon became refractory to treatment, resulting from multiorgan damage, and expired on hospital day 13.

## Introduction

Adult-Onset Still’s disease (AOSD) is a rare, multisystem, autoinflammatory disease noted by the appearance of polyarthritis, pyrexia, high serum ferritin levels, neutrophilic leukocytosis and evanescent rash of unknown etiology. Many other clinical observations are also commonly seen in combination with the previously stated symptoms, including serositis, sore throat, splenomegaly, hepatomegaly, and lymphadenopathy [1]. Since first being characterized and termed by Eric Bywaters in 1971, AOSD has continued to be a difficult disease to diagnose and remains a diagnosis of exclusion due to varying clinical presentations and relative lack of disease-specific markers [2].

Further, AOSD is a relatively rare condition with a documented prevalence rate of approximately 1-34 cases per 1 million people [3]. AOSD commonly affects patients in two separate age ranges-15-25 and 36-46-but it has also been shown to occasionally affect older populations [1]. While AOSD is traditionally not associated with a high mortality rate, conditions such as hemophagocytic lymphohistiocytosis (HLH) can appear secondary to AOSD and are associated with a drastic increase in patient mortality [4]. Secondary HLH involves dysfunctional activation of macrophages and lymphocytes, resulting in overexpression of pro-inflammatory cytokines and is often triggered by pathogens such as Epstein-Barr virus (EBV) and cytomegalovirus (CMV) or inflammatory conditions such as neoplastic disease and autoimmune disorders [1]. In addition, HLH exacerbates many of the common symptoms of AOSD, such as hepatomegaly, serositis, liver dysfunction, renal dysfunction, leukopenia, and anemia [5]. We present the case of a 53-year-old African American woman who presented with AOSD complicated by secondary HLH, was unresponsive to intravenous (IV) steroid treatment, and ultimately progressed to multisystem organ failure and death.

## Case presentation

A 53-year-old female with a past medical history of essential hypertension, herpes simplex virus type 1 infection, obesity, carpal tunnel syndrome of the right wrist, and left knee osteoarthritis status post left total knee arthroplasty presented to the emergency department for complaints of subjective fevers, sore throat, generalized weakness, right-sided low back pain, cough, arthralgias, and myalgias for one week. She presented to her primary care physician with these complaints three days prior to admission and was treated with oseltamivir, ibuprofen, cyclobenzaprine, and azithromycin, but symptoms continued to progress. Tests for rapid streptococcus, coronavirus disease 2019 (COVID-19), and influenza resulted in negative at that time. Notably, she had seen a dermatologist five months prior with complaints of diffuse pruritic rash that was constantly present for over two months. At that time, the dermatologist noted erythematous, edematous papules and plaques over bilateral upper and lower extremities, as well as, dermatographism. She was treated for scabies and was prescribed hydroxyzine and permethrin cream. She had a past surgical history of a total knee arthroplasty three months prior without any complications, a total abdominal hysterectomy with bilateral salpingectomy three years prior, as well as a tonsillectomy and adenoidectomy 20 years prior. She had no known allergies. Other medications included acetaminophen, acyclovir, amitriptyline, cholecalciferol, losartan, hydrochlorothiazide, diclofenac, famotidine, and docusate sodium. Social history included no recent travel, no known exposure to sick contacts, no history of tobacco or drug use, and occasional alcohol consumption. She worked as a school bus driver. Her family history was notable for maternal breast cancer and paternal Alzheimer’s dementia and heart disease but no autoimmune disease. A review of systems was negative for chest pain, shortness of breath, cough, abdominal pain, diarrhea, constipation, nausea, vomiting, and dysuria.

On admission, she was noted to be tachycardic, hypertensive, tachypneic, and febrile up to 39.4ºC. Her oxygen saturation was within normal limits on room air. Physical exam was notable for an ill-appearing individual with crackles at bilateral lung bases. Laboratory studies on admission revealed an elevated white blood cell (WBC) count with neutrophilia, normocytic anemia, hypokalemia, and elevated aspartate aminotransferase, proteinuria, and trace ketones and leukocytes in the urine (Table 1).

**Table 1:** Laboratory values throughout the hospital course BUN: Blood urea nitrogen; AST: Aspartate aminotransferase; ALT: Alanine aminotransferase; eGFR AA: Estimated glomerular filtration rate for African Americans

Lab Results	Reference Range	Day													
		1	2	3	4	5	6	7	8	9	10	11	12	13	14
WBC	4.5 - 11.0 10^3^/uL	13.82	14.81	15.54	19.63	22.95	23.57	22.53	27.9	28.91	19.19	40.5	37.23	31.05	25.88
Hemoglobin	13.5 - 17.5 gm/dL	11.2	10.8	10.4	11.1	10.9	10.4	10.1	10.6	10.5	9.5	10.4	11.4	12.3	14
Hematocrit	40 - 51 %	36.2	34.6	32	33.6	33.6	31.7	31.8	32.3	32.2	29.1	31.1	34.6	37.8	45.1
Platelets	130 - 400 10^3^/uL	337	333	341	373	332	371	349	356	330	258	268	293	231	192
Granulocyte	40 - 60 %	86.2	81.2	87.4	91.1	91.2	88.8	88.6	92	89.4	90	86	92	93	90
Lymphocyte	20 - 40 %	8.5	11.5	6.8	5.3	4	4.2	4	5	2.6	2	5	3	1	6
Monocyte	4 - 8 %	4.4	6.1	4.2	2.5	2.8	4	2.2	2	2	0.5	1	1	0	0
Eosinophil	1 - 3 %	0.1	0.4	0.5	0.2	0.3	0.8	1.5	1	2.5	2.5	1	1	0	1
Basophil	0 - 1 %	0.3	0.3	0.3	0.3	0.3	0.2	0.4	0	0.3	0	0	0	0	0
Sodium	135 - 146 mmol/L	136	135	135	133	131	131	129	-	126	128	127	124	120	117
Potassium	3.6 - 5.2 mmol/L	3.3	3.7	3.1	3.9	3.9	3.8	3.4	-	4		4.3	5	5.3	8.8
Chloride	96 - 110 mmol/L	99	99	100	97	95	95	94	-	92		94	93	90	89
Calcium	8.4 - 10.3 mg/dL	9.7	8.6	9.4	9.4	9.1	8.9	8.7	-	8.8	7.6	8.3	8.2	7.7	6.2
CO2	24 - 32 mmol/L	22	23	22	22	20	22	24	-	19		18	16	12	8
Glucose	65 - 99 mg/dL	129	107	139	152	117	127	124	-	112	97	130	113	125	406
BUN	7.0 - 25.0 mg/dL	12	13	10	9	14	19	20	-	25	29	49	72	93	74
Creatinine	0.70 - 1.40 mg/dL	0.8	0.8	0.7	0.8	1.4	1.4	1.6	1.5	1.8	2.2	3	3.9	6.2	4.2
Albumin	3.4 - 5.0 g/dL	2.9	2.5	2.3	2.2	-	-	-	-	1.8	1.7	1.6	1.6	1.6	1.1
Total Protein	6.0 - 8.0 g/dL	7.3	5.7	6.2	6.5	-	-	-	-	6.1	5	5.3	5.2	4.8	5
Total Bilirubin	0.2 - 1.2 mg/dL	0.8	0.9	0.7	0.7	-	-	-	-	0.6	0.5	0.6	0.6	1.4	1.1
Alkaline Phosphatase	37 - 153 U/L	128	156	158	151	-	-	-	-	175	157	165	182	177	191
AST	10 - 35 U/L	52	120	62	68	-	-	-	-	131	182	313	423	698	1,315
ALT	6 - 29 U/L	42	95	69	67	-	-	-	-	82	99	159	192	240	367
Anion Gap	8 - 16 mmol/L	15	13	13	14	16	14	11	-	15	18	15	15	18	22
eGFR AA	>90.0 mL/min/1.73m^2 ^	>60	>60	>60	>60	49.5	49.5	42.1	45.5	36.5	28.7	19.7	14.3	10.4	16.9

Lactic acid was normal, and the respiratory viral panel was negative. A computed tomography (CT) scan of the abdomen and pelvis revealed bibasilar subsegmental atelectasis and a 1.1-centimeter (cm) hypoattenuating focus in the right hepatic lobe. CT angiography of the chest was negative for pulmonary embolism, and the chest X-ray was unremarkable. The electrocardiogram revealed sinus tachycardia. She was given a dose of vancomycin and piperacillin-tazobactam in the emergency department.

On day 2 of her hospitalization, the infectious disease service was consulted and suggested that symptoms could be viral and therefore, antibiotics were held. While her WBC count steadily increased, the patient continued to have a fever as high as 39.5ºC (Table 2).

**Table 2:** Vital signs throughout the hospital course

Day	Temperature (°C)	Pulse	Respiratory Rate	Blood Pressure	SpO2
						1	39.56	109	24	158/83	97%
2	36.78	92-133	17-19	141/68	91-100%
3	39.50	99-131	16-20	139/73	96-100%
4	37.44	131	20	135/65	93%
5	38.72	131	16	128/67	93%
6	37.61	128	18	161/75	98%
7	37.72	127	18	132/63	94%
8	37.00	98	18	117/56	94%
9	36.94	107	18	142/67	96%
10	39.72	120	22	126/60	95%
11	37.33	110	22	129/65	97%
12	38.17	114	18	134/77	95%
13	39.00	117	20	129/62	92%
14	38.00	109	35	153/89	92%

Magnetic resonance imaging (MRI) of her thoracic and lumbar spine was recommended, which revealed degenerative changes of the lower lumbar levels, most pronounced at the right L4-L5 facet joint where there was hypertrophy and subchondral cyst formation. In addition, there was edema and enhancement about the right facet joint and 1.2 cm of extra-articular peripherally enhancing fluid collection. Over the next few days, high fevers and tachycardia continued to persist. In addition to increasing WBCs, blood tests revealed elevated erythrocyte sedimentation rate (ESR), C-reactive protein (CRP), and procalcitonin. HIV and repeat COVID-19 tests remained negative, and blood cultures revealed no growth. Intravenous vancomycin and ceftriaxone were restarted three days after admission. At that time, interventional radiology was consulted for aspiration of possible abscess, but this was deemed too small for aspiration and technically difficult.

On hospital day 5, ceftriaxone was broadened to cefepime. The patient’s fevers and tachycardia persisted while WBCs, ESR, and CRP continued to rise, and creatinine doubled from baseline. Antinuclear antibody testing was acquired, which later resulted in a negative. Repeat lactic acid was normal. Orthopaedic spinal surgery was consulted and deemed the possible fluid collection unlikely to be the source of sepsis. Bilateral knee X-rays were acquired, which showed stable arthroplasty on the left with a small effusion, as well as, tricompartmental osteoarthritis with effusion on the right. A nuclear medicine indium-111 WBC scan was performed, which did not identify a source of infection.

On hospital day 6, back pain had resolved, creatinine levels were stable, and the patient was producing urine. However, laboratory tests continued to display increasing WBCs. A transthoracic echocardiogram revealed a small circumferential pericardial effusion but was otherwise unremarkable. Bilateral lower extremity deep venous thrombosis ultrasounds were negative. A chest X-ray was acquired, which was concerning for a new left lung base opacity with pleural effusion. Azithromycin was started at this time. Respiratory culture, legionella urine antigen, serology for mycoplasma pneumoniae, and polymerase chain reaction (PCR) to detect cytomegalovirus DNA and HIV1 DNA and RNA resulted in negative. An EBV antibody panel was also collected, which revealed an elevated viral capsid antigen (VCA)-immunoglobulin (Ig)G antibody, a normal VCA-IgM antibody, and elevated early antigen and nuclear antigen IgG antibodies. Other laboratory studies revealed normal thyroid function and a positive rheumatoid factor. WBC count and procalcitonin continued to rise. Blood cultures remained negative. The infectious disease team recommended discontinuing vancomycin, cefepime, and azithromycin and switching to oral levofloxacin. Given concern for a parapneumonic effusion, a repeat CT chest without contrast was ordered, revealing pulmonary edema, bilateral pleural effusions, mild pericardial effusion, and small consolidative opacities at the lung bases possibly reflecting atelectasis.

On hospital day 9, the patient was noted to be hallucinating, and levofloxacin was discontinued as a possible etiology. She was experiencing diarrhea, so collection and testing for *Clostridium difficile* (C. diff) was performed. The rheumatology service was consulted and noticed an evanescent rash during a febrile episode of up to 39.4ºC. At this time, there was a concern for AOSD, and initiation of corticosteroids was discussed as a treatment option following the exclusion of *C. difficile* infection. Additional laboratory tests revealed unremarkable anti-cyclic citrullinated peptide antibody, hepatitis viral panel, B-type natriuretic peptide, creatinine kinase, immunoglobulins, serum protein electrophoresis, and immunofixation. Ferritin, interleukin-2 receptor, D-dimer, fibrinogen, and lactate dehydrogenase levels were markedly elevated (Table 3).

**Table 3:** Miscellaneous laboratory values LDH: Lactate dehydrogenase; CRP: C-reactive protein; ESR: Erythrocyte sedimentation rate

Test	Reference Range	Result
Ferritin	12 - 150 ng/mL	>26,000
D-Dimer	<0.50 mg/L FEU	>33
Fibrinogen	200 - 400 mg/dL	650
LDH	135 - 214 U/L	1642
CRP	<10.0 mg/L	473.1
ESR	<20.0 mm/hr	>120
Procalcitonin	<0.1 ng/mL	9.2
Haptoglobin	40 - 200 mg/dL	444

On hospital day 10, the patient continued to have a fever up to 39.7°C with increasing WBC count and transaminases. The renal function continued to decline despite intravenous fluid resuscitation. Anti-neutrophilic cytoplasmic antibody testing was ordered and resulted in a negative. After the patient’s *C. difficile* testing resulted as negative, IV methylprednisolone was administered at 125 milligrams every six hours.

The patient was noted to feel much improved after initiation of IV methylprednisolone. She was also afebrile for 24 hours, but since creatinine continued to rise, nephrology was consulted. A repeat MRI of her lumbar spine re-demonstrated facet joint edema improved from the prior. Previously described paraspinal collections were not well delineated. However, on hospital day 11, the patient became tachycardic with a low-grade fever. WBC remained increased but stable. Urine studies again revealed proteinuria.

On hospital day 12, hepatology was consulted for increasing transaminases. To rule out primary biliary cholangitis and autoimmune hepatitis, anti-mitochondrial and anti-smooth muscle antibodies, respectively, were ordered and resulted in negative. However, alpha-1-antitrypsin and ceruloplasmin were mildly elevated. Lactic acid and procalcitonin were markedly elevated. Creatinine continued to rise and urine output began to decline despite high-dose diuretics, and the patient now required two liters of oxygen by nasal cannula. At this time, the patient continued to fever up to 38.9°C and was transferred to the ICU. Rheumatology recommended initiation of intravenous immunoglobulin 1g/kg, pulse dose steroids for three days, and subcutaneous anakinra. Continuous veno-venous hemodiafiltration (CVVH) was also initiated. During trialysis line placement, the patient showed signs of respiratory distress and agonal breathing and was initiated on BiPAP® (a noninvasive ventilation machine), which resulted in no improvement, so the patient was emergently intubated. Hematology was consulted for consideration of hemophagocytic lymphohistiocytosis, and a bone marrow biopsy was scheduled for the next morning; however, overnight, the patient became intolerant to CVVH due to hypotension, and she was on the maximum dose of norepinephrine, epinephrine, and vasopressin. Upper and lower extremity DVT studies revealed nonocclusive thromboses of the right common femoral and femoral veins and an occlusive thrombus of the left posterior tibial vein. Despite interventions, the patient continued to decline and unfortunately expired on hospital day 13.

An autopsy revealed pathology in the heart, lungs, kidneys, and liver. The heart displayed signs of severe, diffuse pericarditis with cardiomegaly (400g), left ventricular hypertrophy (2.3 cm), and coronary artery atherosclerosis. She was also suffering from bilateral pulmonary congestion and acute bronchopneumonia of the right lower lobe and the left upper and lower lobes. The kidneys exhibited bilateral, acalculous hydronephrosis and mild focal glomerular sclerosis. Liver pathology revealed mild portal and parenchymal fibrosis. Together with the potentially acute effects of the clinical course, the findings of chronic pericarditis, liver fibrosis, and kidney disease led to the final determination that the patient died from multi-organ failure secondary to AOSD.

## Discussion

AOSD is a rare, idiopathic, inflammatory condition characterized by a fever of acute onset that often resists initial treatment with common antipyretic medication. Along with the unrelenting fever, patients present with a characteristic evanescent rash, arthritis, lymphadenopathy, splenomegaly, and pharyngitis. To make a diagnosis of AOSD, the Yamaguchi set of criteria is often used for its superior sensitivity (96.2%) [6-8] and includes both a major criteria category of: 1) elevated fever lasting over one week, 2) arthralgias or arthritis lasting two weeks at minimum, 3) evanescent rash, and 4) leukocytosis, and a minor criteria category of: 1) abnormal liver function tests, 2) hepatomegaly or splenomegaly, 3) sore throat, 4) lymphadenopathy, and 5) negative antinuclear antibody or rheumatoid factor). To diagnose AOSD using these criteria, infectious and neoplastic diseases must be ruled out, and at least five of the features must be present, with a minimum of two major criteria [8]. In the case presented, malignancy was lower on the differential as a cause of her symptoms since no masses were identified and laboratory tests did not suggest activity specific to neoplastic disease. While infection was considered part of the initial differential diagnosis, extensive radiographic testing and cultures failed to produce infectious etiology. In addition, a possible spinal abscess on MRI was ruled out by orthopedic surgery as the source of infection and deemed too small for aspiration by interventional radiology. Further, a spinal abscess being a source of infection does not fit her clinical picture as her back pain resolved spontaneously. Similarly, a chest X-ray was concerning for a left lung base opacity with pleural effusion, but a respiratory viral panel and all sputum cultures were negative, and she was covered broadly with antibiotics and treated for possible pneumonia appropriately without improvement, effectively ruling the lungs as an unlikely source of infection. While each localized source of infection was ruled out, many general tests also suggested infection was not the cause of her symptoms, and a nuclear medicine indium-111 white blood cell scan was performed with no scintigraphic finding to suggest a source of infection. Further, viral infections would be unlikely given the neutrophilic predominance of her leukocytosis as well as negative tests for COVID-19, influenza, HIV, CMV, EBV, and viral hepatitis.

**Figure 1:**
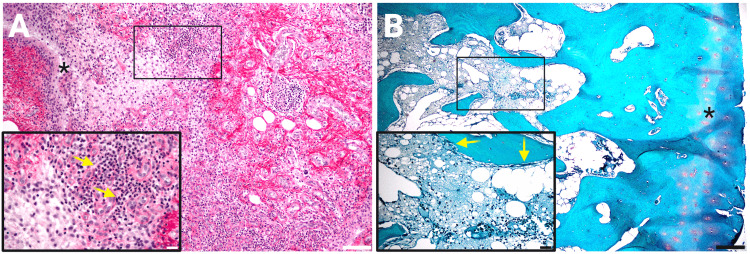
High neutrophil infiltration in synovial tissue and subchondral lesions of the medial femoral condyle. (A) 100x photomicrograph (bar = 100 μm) of synovial tissue stained by picrosirius technique with marked lymphocytic aggregates, fibrous exudate, and hypervascularity below the hyperplastic intima (asterisk) with widespread neutrophil infiltrates (400x inset; yellow arrows; bar = 20 μm). (B) 40x photomicrograph (bar = 200 μm) of medial condyle stained with Safranin O (red) and light green shows chronic degenerative features such as matrix and proteoglycan loss at the cartilaginous surface (asterisk) with fissures, tidemark duplication, and sclerosis of subchondral trabeculae filled with macrophages and lymphoplasmic cell aggregates and prominently lined by osteoclasts (200x inset; yellow arrows; bar = 50 μm).

Since evidence indicated a lack of neoplastic or infectious disease and the patient met the major Yamaguchi criteria of persistent fever for over a week, evanescent rash, leukocytosis, and arthritis with minor criteria of abnormal liver function, negative antinuclear test, and sore throat, the patient most likely had AOSD. In addition, this patient developed common laboratory findings seen in AOSD, such as highly elevated serum ferritin, CRP, and ESR levels as well as transaminitis and anemia. Furthermore, neutrophilic leukocytosis, which is commonly seen with cases of AOSD, was observed in laboratory tests and validated by the extensive presence of neutrophilic infiltrates on histological samples of synovium and subchondral bone from the femoral condyles collected during total knee arthroplasty for osteoarthritis (Figure 1).

Finally, while the highly sensitive Yamaguchi’s criteria are most commonly used, clinical findings also met Fautrel’s criteria, which have the highest specificity (98.5%) for AOSD [9]. Fautrel’s criteria elaborates on Yamaguchi’s criteria by including two novel diagnostic markers for AOSD: serum ferritin and glycosylated ferritin levels. Additionally, it does not require any infectious, systemic, or malignant conditions to be ruled out before making a diagnosis of AOSD. Fautrel’s criteria require four out of six major criteria for a diagnosis of AOSD: 1) spiking fever over 102.2°F, 2) arthralgias, 3) transient erythematous rash, 4) pharyngitis, 5) polymorphonuclear cells ≥80%, 6) glycosylated ferritin ≤20%) or three of these major criteria plus two minor criteria: 1) maculopapular rash and 2) leukocytes ≥10,000/mm^3^ [9]. This patient met the major criteria of spiking fevers, arthralgias, pharyngitis, polymorphonuclear cells ≥80%, and the minor criteria of maculopapular rash and leukocytes ≥10,000/mm3. Overall, the combination of meeting both Yamaguchi’s and Fautrel’s criteria paired with her final autopsy overwhelmingly supports a diagnosis of AOSD.

Given this patient's previous history within the last 12 months of dermatographism, a rash characterized by erythematous, edematous papules and plaques over bilateral upper and lower extremities, and her history of knee osteoarthritis (KOA), we predict that she had been living with the more minor manifestations of the disease well before hospitalization. This hypothesis is supported by her neutrophilia status and histology of fragments from the suprapatellar synovium and medial femoral condyle obtained for consented osteoarthritis research during total knee arthroplasty four months before death that showed excessive neutrophilic infiltrates throughout, which are not typical of mechanical or primary KOA (Figure 1). These unusual findings led to this investigation and the discovery of an AOSD diagnosis with an unfortunate clinical course and outcome. Given the totality of these findings and a growing body of literature suggesting that infections can “activate” AOSD flare-ups and result in a previously controlled AOSD requiring hospitalization [1], we predict that she became infected with EBV, likely from her job as a school bus driver, which triggered a more severe manifestation of an otherwise controlled AOSD status. This hypothesis is supported by positivity for early antigen IgG antibodies against EBV, which has been known to trigger AOSD [10,11]. However, it remains unclear how viruses and other pathogens are capable of triggering AOSD besides generally stimulating a pro-inflammatory state.

Treatment for AOSD typically involves the administration of various anti-inflammatory medications, including non-steroidal anti-inflammatory drugs (NSAIDs), high-dose corticosteroids, and various inhibitors of pro-inflammatory cytokine cascades [12]. While this treatment regimen often results in a regression of symptoms, some cases of AOSD are refractory to treatment and are complicated by conditions such as secondary hemophagocytic lymphohistiocytosis (HLH). HLH shares many symptoms with AOSD and is characterized by the secretion of a cytokine storm resulting from a dysregulation in monocyte activation to the predominance of a cytotoxic macrophage phenotype [5]. The onset of HLH secondary to AOSD can not only cause a patient to be more resistant to treatment but also raise the mortality rate to an alarming 41% [13]. A diagnosis of HLH involves fulfilling the criteria of observed symptoms in the validated HLH-2004 set and/or by calculating an Hscore [14]. The HLH-2004 expands on the HLH-94 set of criteria: 1) fever, 2) splenomegaly, 3) bicytopenia, 4) hypertriglyceridemia and/or hypofibrinogenemia, and 5) hemophagocytosis by adding three more criteria: 1) low/absent NK-cell-activity, 2) hyperferritinemia, and 3) high-soluble interleukin-2-receptor levels. A patient’s HLH-2004 set that includes at least five of the eight criteria certifies an HLH diagnosis with a sensitivity of 91% and specificity of 93% [15]. In the presented case, the patient met four of the eight HLH-2004 criteria: 1) fever, 2) hyperferritinemia, 3) high-soluble interleukin-2-receptor levels, and 4) hypertriglyceridemia; however, natural killer (NK) cell activity was never measured, making the final score unreliable. Thus, in the present case, an Hscore was calculated, which consists of many of the same criteria from the HLH-2004 criteria, but excludes NK cell activity and weights the lab findings based on how abnormally high or low they are.

Originally, an Hscore of 182 was calculated on site a few days before death, which corresponds to a 70-80% probability that she developed secondary HLH. However, based on a comprehensive inclusion of relevant parameters extracted from her chart by our group, the Hscore had progressed to 212 by the time of death, which correlates to a 93%-96% probability of secondary HLH status. While HLH is typically a disease occurring in children or adults with a family history (primary HLH), secondary HLH can manifest in conjunction with the onset of other cases of autoimmune or infectious diseases [3] such as AOSD. For this reason, considering the overlapping symptomology of AOSD and HLH and the elevated Hscore recorded, we suggest that the present case could represent HLH presentation secondary to AOSD, as opposed to primary HLH. This also explains why leukopenia, a distinguishing feature of primary HLH, was absent in this case and leukocytosis, a hallmark of AOSD, was present throughout her hospital course. Finally, although the typical cytological features of the bone marrow seen in HLH, such as diffuse histiocytic infiltration, histiocyte hyperplasia, and variable cytotoxic T-cell numbers ± hemophagocytosis [16] were not observed or reported from autopsy material, HLH cannot be ruled out from this finding alone given the low sensitivity and specificity that these particular findings contribute to HLH diagnosis [17].

As previously noted, interventions for AOSD involve the administration of high-dose corticosteroids and various cytokine inhibitors. In this case, treatment involved the initial administration of IV methylprednisolone 125 milligrams every six hours, followed by subcutaneous administration of the interleukin-1 receptor antagonist, anakinra, and intravenous immunoglobulin (IVIG). Although the patient was mostly unresponsive to this combinatorial treatment based on laboratory tests, she became afebrile for 24 hours and reported feeling much better after the initiation of steroid treatment. Thus, it is possible that her symptoms were responsive to treatment, but her underlying multiorgan damage had progressed too far by that point to make a complete recovery.

In other cases of AOSD, patients who are unresponsive to treatment with high-dose steroids and anakinra are treated with emapulumub, a novel approach to AOSD using an inhibitory monoclonal antibody against interferon-gamma. In one case, an AOSD patient unresponsive to empiric treatment was treated with emapalumub, which resolved all symptoms and abnormal lab values, including fever, arthralgia, liver dysfunction, and splenomegaly [18]. The success of emapalumub and other interferon gamma antagonists, which had not been used for AOSD until this point [19], could possibly provide early evidence for a new treatment approach for AOSD patients unresponsive to empiric treatments. Finally, tocilizumab, an antibody against the interleukin-6 receptor, is also effective in refractory cases of AOSD, with one case series showing that five of eight patients entered stable remission after treatment. Out of the remaining three patients, only one relapsed, while the last two had to pause treatment due to infection [20]. Coupled with a comprehensive and definitive diagnostic strategy for primary AOSD with secondary conditions such as HLH, understanding why these patients are unresponsive or refractory to treatment will be paramount to the success of novel individualized approaches to care for AOSD patients.

## Conclusions

This report presents a case of AOSD with reactive HLH that was refractory to the current standard of treatment. Further evidence is needed to elucidate the mechanism by which pathogens can trigger AOSD symptoms and why some patients are refractory to current treatments despite excellent symptom control in many AOSD patient subpopulations.
